# Obsessive–compulsive disorder onset and clinical course in the context and treatment of pineal region germinoma and obstructive hydrocephalus: a case report

**DOI:** 10.3389/frcha.2026.1718502

**Published:** 2026-02-03

**Authors:** Andrea Saliba, S. Evelyn Stewart

**Affiliations:** 1 British Columbia Children’s Hospital, Vancouver, BC, Canada; 2 British Columbia Children’s Hospital Research Institute, Vancouver, BC, Canada; 3 Department of Psychiatry, Faculty of Medicine, University of British Columbia, Vancouver, BC, Canada

**Keywords:** cerebrospinal fluid diversion, cortico-striat-o-thalamo-cortical circuits, hydrocephalus, neuroimaging, obsessive–compulsive disorder, pediatric, pineal germinoma

## Abstract

Obsessive–compulsive disorder (OCD) secondary to structural brain pathology is rarely described in pediatric populations. This single-case report describes a child with a pineal germinoma and obstructive hydrocephalus who developed prominent obsessive–compulsive symptoms, highlighting a potentially reversible neurobiological contributor to atypical pediatric OCD. The case illustrates the temporal association between symptom evolution, neurosurgical and oncologic management, and subsequent changes in obsessive–compulsive behaviors, contributing to understanding the role of cortico-striato-thalamo-cortical (CSTC) circuit disruption in secondary OCD. A 15-year-old boy initially presented with a 12-month history of mild generalized anxiety and intermittent insomnia, which progressed over the subsequent 8 months to severe obsessive thoughts and compulsive behaviors, including checking and repetition, significantly impairing sleep and food intake. Two months prior to tertiary referral, he developed progressive neurological symptoms, including afternoon headaches, blurred vision, gait instability with falls, polyuria, enuresis, and one witnessed episode of loss of consciousness. A neurological examination demonstrated Parinaud's syndrome, hyperreflexia with ankle clonus, extensor plantar responses, past-pointing, and papilledema. Neuroimaging (CT and MRI) revealed a pineal mass causing aqueductal obstruction with hydrocephalus, periventricular edema, and a suspected vestibular schwannoma. Endoscopic third ventriculostomy with biopsy and external ventricular drainage confirmed germinoma and relieved the cerebrospinal fluid (CSF) obstruction, with postoperative imaging demonstrating restored CSF flow. The patient subsequently received chemotherapy and focal radiotherapy. Following CSF diversion, the patient’s caregivers reported a marked reduction in compulsive rituals and improved sleep, with further attenuation of OCD symptoms during oncologic treatment. Residual anxiety and mild obsessive–compulsive symptoms were managed with adjunctive selective serotonin reuptake inhibitor therapy. At the 12-month follow-up, standardized assessments indicated subclinical to mild OCD, and the patient had returned to full academic participation. This case underscores the importance of considering hydrocephalus-related CSTC circuit disruption as a potential, and occasionally reversible, contributor to pediatric OCD. Recognizing hydrocephalus-associated obsessive–compulsive symptoms may significantly influence both psychiatric and neurosurgical management. Clinicians evaluating atypical or treatment-refractory pediatric OCD should consider neuroimaging to identify underlying structural causes, emphasizing the value of interdisciplinary collaboration in optimizing outcomes.

## Introduction

Obsessive–compulsive disorder (OCD) is increasingly understood as a network disorder arising from dysfunction within cortico-striat-o-thalamo (CSTC) circuits. Early positron emission tomography studies demonstrated that successful pharmacological or behavioral treatment of OCD reduces metabolic hyperactivity in the caudate nucleus, a central node of the CSTC loop (1). Deep transcranial magnetic stimulation for OCD in adults has shown positive results from stimulation of a central part of the CSTC (2). Diffusion tensor imaging has identified microstructural abnormalities in fronto-striatal white matter tracts not only in individuals with OCD but also in their unaffected first-degree relatives, suggesting a heritable vulnerability in these pathways (3). Moreover, results from the ENIGMA OCD Working Group’s (4) meta-analyses reported increased thalamic volume in pediatric but not adult OCD.

Pediatric white matter tracts continue to mature throughout adolescence, rendering them especially susceptible to mechanical and inflammatory insults during critical developmental windows (5). Activity-dependent myelination has emerged as a key mechanism for refining neural connectivity, and disruptions to this process can have long-lasting effects on network function (6). Mechanical stress on periventricular white matter, particularly involving CSTC circuits, can precipitate obsessive–compulsive behaviors (7). OCD has been reported following acute inflammatory white-matter insults such as acute disseminated encephalomyelitis (8), implicating CSTC disruption as a mechanism for compulsive behaviors.

Thus, hydrocephalus-induced injury to periventricular tracts may not only trigger acute OCD symptoms but also influence the trajectory of CSTC circuit maturation in children, underscoring the importance of early identification and intervention (3, 5). The present case of OCD onset and severity resolution in direct temporal relation to pineal region germinoma and hydrocephalus further supports the notion that mechanical and inflammatory perturbation of CSTC pathways can precipitate and relieve obsessive–compulsive symptomatology.

## Case description

A 15-year-old boy presented with a long-standing history of mild generalized anxiety disorder (GAD) and developed OCD symptoms with concurrent neurological symptoms. He was born at term via emergency cesarean without complications and had no prior attention, behavioral, or major mood concerns. His developmental and school performance (As/Bs) were normal, with up-to-date immunizations and a known tree-nut allergy. There was no family history of OCD, pediatric malignancy, brain tumors, seizures, or genetic syndromes.

**Figure 1:**
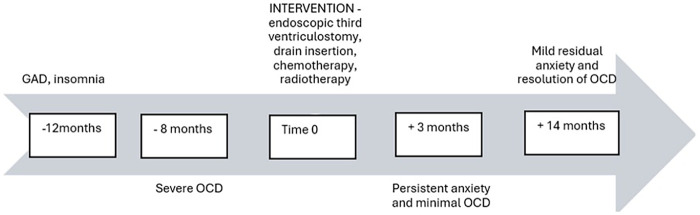
The clinical course timeline with relevant descriptive data.

Over the year preceding presentation, he experienced mild generalized anxiety and intermittent insomnia, accompanied by food restriction and weight loss. He had an 8-month history of nightly, 3-and-a-1⁄2-h rituals that included stepping down stairs always on the left foot, repeated coughing, re-closing doors/toilet seats, and fixed toileting sequences. He described these as a “checklist” that relieved distress and facilitated sleep; he believed that prevention of ritual completion could delay sleep by up to 9 h. Two months prior to referral to tertiary services, he developed progressive afternoon headaches, blurry vision, intermittent diplopia, difficulty focusing, gait unsteadiness with falls, polyuria, enuresis, and one witnessed episode of loss of consciousness. He presented to the hospital after two falls and a brief loss of consciousness. Figure 1 shows the clinical course timeline.

## Diagnostic assessment

**Figure 2:**
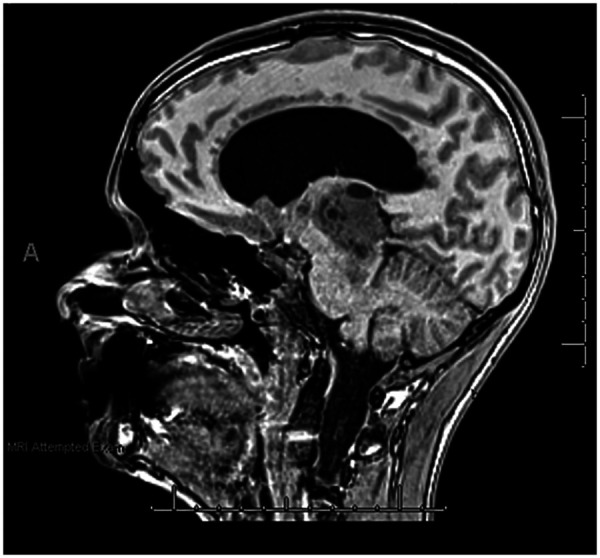
Sagittal T1 Magnetization-Prepared RApid Gradient Echo (MPRAGE) MRI of the patient’s head. The image was taken when the OCD was the most severe (by both youth and parent report).

A neurological examination revealed vertical gaze palsy, hyperreflexia with ankle clonus, upgoing plantars, positive past-pointing, and papilledema. CT demonstrated a 4.5 cm pineal mass obstructing the aqueduct and causing hydrocephalus; MRI showed a predominantly solid 43 mm × 41 mm × 44 mm lesion with cystic foci, thalamic involvement, and periventricular edema as seen in Figure 2. He was diagnosed with pineal germinoma with hydrocephalus, a left internal auditory canal lesion with presumed acoustic schwannoma, Parinaud's syndrome, and papilledema.

He underwent endoscopic third ventriculostomy, biopsy, and external ventricular drain insertion; no resection was attempted. An intraoperative smear and immunohistochemistry confirmed germinoma; postoperative imaging showed decreased ventricular size and restored cerebrospinal fluid (CSF) flow without metastases. He received chemotherapy followed by radiotherapy.

**Figure 3:**
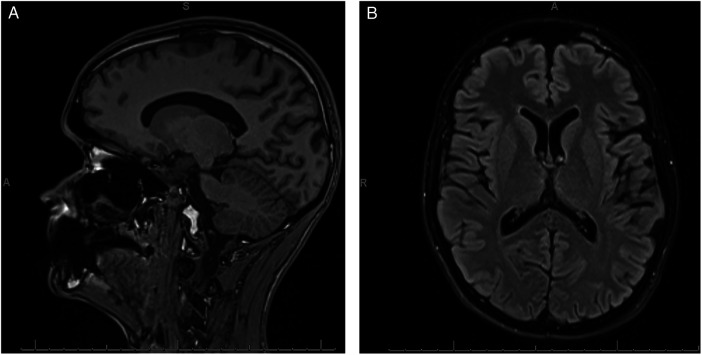
**(A,B)** Axial and sagittal T1 MPRAGE MRI of the patient’s head. The image was taken 14-month postsurgical intervention when the OCD was subclinical (youth report).

Following CSF diversion and prior to chemotherapy, the patient’s parents reported slight improvements in his OCD symptoms, together with consolidated sleep, reduced auditory “muffling,” decreased nocturnal urinary frequency, and improved neurological symptoms. They also observed that his evening compulsions had markedly lessened once chemotherapy began. Three months postoperatively, he started sertraline before switching to fluvoxamine for persistent generalized anxiety symptoms and minimal OCD symptoms. He exhibited mild residual OCD symptoms; however, their severity was markedly reduced compared to the period during which he had hydrocephalus. At 12 months postdiagnosis, on the Child Obsessive-Compulsive Impact Scale-Revised (COIS-R), he had a daily living skills mean of 0.8, social mean of 1.3, family/activities mean of 0.9, and school mean of 1, in keeping with “just a little” impact. The COIS-R parent-rated total was 35 out of 99. Application of the Family Accommodation Scale for Obsessive-Compulsive Disorder (FAS-OCD) resulted in a FAS mean of 0.2 and a FAS total of 4. At 14-month postdiagnosis, the patient's self-rated Children’s Yale-Brown Obsessive Compulsive Scale (CY-BOCS) total (sum of the first 10 severity items) was 4, with an additional extension-question score of 1, consistent with subclinical symptom severity. See figure 3 for MRI scan. Academically, he resumed school without accommodations, reporting only mild residual anxiety and resolution of OCD.

## Discussion

Although primary OCD remains a possibility, the close temporal association between symptom onset and tumor development and symptom improvement following CSF diversion and subsequent treatment suggest a secondary OCD mechanism (3). This report highlights obstructive hydrocephalus as a potentially reversible cause of obsessive–compulsive behaviors in children. By exerting pressure on periventricular white matter, hydrocephalus may mimic the primary CSTC circuit dysfunction typical of OCD (7, 9), and timely CSF diversion can lead to marked symptomatic improvement without prolonged psychiatric treatment. Recognizing reversible, hydrocephalus-induced OCD can significantly alter both psychiatric and neurosurgical management in pediatric populations. Clinicians evaluating atypical or treatment-refractory pediatric OCD could consider neuroimaging to identify underlying structural causes.

The case further suggests that secondary OCD due to hydrocephalus shares mechanistic overlap with primary CSTC circuit dysfunction, emphasizing the need for integrated neuropsychiatric and neurosurgical care (6, 9). Multidisciplinary assessment can optimize both neurological and psychiatric outcomes by addressing the root structural pathology alongside symptomatic management, as demonstrated by the critical role of psychiatric monitoring, pharmacotherapy adjustments, and collaboration with neurosurgical and oncological teams in this case.

Future studies employing high-resolution connectomic analyses and longitudinal imaging will help delineate the timeline of CSTC circuit injury and recovery following CSF diversion (10). Integrating detailed neurodevelopmental assessments with neuropsychiatric outcome measures may inform standardized screening and intervention protocols, ultimately enhancing care for children with hydrocephalus-related and primary forms of OCD.

## Data Availability

The original contributions presented in the study are included in the article, further inquiries can be directed to the corresponding author.
